# Stakeholders’ Perceived Barriers and Successes of Quality Improvement Programs for Patients With Diabetes: Comment on "Quality and Performance Measurement in Primary Diabetes Care: A Qualitative Study in Urban China"

**DOI:** 10.34172/ijhpm.2023.7831

**Published:** 2023-12-25

**Authors:** Ana Neumann

**Affiliations:** Department of General Practice and Dental Public Health, School of Dentistry, The University of Texas Health Science Center at Houston, Houston, TX, USA

**Keywords:** Diabetes, Quality of Care, Qualitative Evaluations, China, Primary Healthcare

## Abstract

Rasooly et al performed a qualitative evaluation to characterize the experiences of 26 stakeholders with the implementation of diabetes-related quality and performance measures delivered in primary healthcare to patients with diabetes in metropolitan China. Results from this cross-sectional investigation identified relevant gaps in primary care delivery for people with diabetes from one major center in China. As diabetes is a prevalent condition worldwide, lessons learned from this research can be useful to guide, refine, and improve quality measurement evaluations in primary care in China and other countries. In this commentary, we comment on the strengths and weaknesses of the study, suggest future directions, and discuss how lessons learned from this research can be helpful to guide, refine, and improve the quality measurement of diabetes care in other countries.

## Introduction

 The World Health Organization (WHO) describes the quality of care as “the degree to which health services for individuals and populations increase the likelihood of desired health outcomes and are consistent with evidence-based professional knowledge.”^1^ The development, implementation, and reporting of quality measures is an international policy issue. As guidance for measure development, The Institute of Medicine (IOM) established six domains of the healthcare system: safe, effective, patient-centered, timely, efficient, and equitable.^2^ While applying the IOM aims and the need to evaluate the quality of care for people with diabetes is global, countries will differ on the weight given for each of the six domains.

 In their research, Rasooly et al^3^ sought to understand the stakeholders’, patients, physicians, and policy-makers, experiences and opinions of quality measures implemented to evaluate the primary care received by patients with diabetes in Shanghai, a large metropolitan center in China, and an early implementation site of quality evaluation of primary care for diabetics. Their research study answered two main questions: (*a*) what is the current state of quality and performance measurement in primary diabetes care, and (*b*) what are the facilitators and barriers to their implementation.

 The reform of the Chinese healthcare system resulted in nearly 96% of the population being covered by some form of health insurance, and primary healthcare doctors are often the first point of contact for patients seeking diabetes treatment. Despite the advances resulting from the reforms, there are significant gaps in the quality of primary care, driving patients to seek care in specialized or tertiary centers. Challenges faced by primary care providers in China include decreased patient volume, and a lack of integrated electronic health records that could integrate primary, secondary, and tertiary care providers.

 The authors used the increasing hospitalizations of diabetes-associated complications as a proxy outcome indicator for the quality of care provided to people with diabetes. They highlighted the urgency to evaluate care, identify gaps, and design interventions that will ultimately strengthen the quality of primary care provided for people with diabetes. Interviews results were presented using ten constructs across the five domains of the Consolidated Framework for Implementation Research: Process, inner setting, outer setting, individual, and intervention characteristics.

## Challenges and Opportunities With Defining and Implementing Diabetes Quality of Care Measures

 Across all five Consolidated Framework for Implementation Research domains, several challenges are evident: First, the implementation of quality measures developed according to the national strategic plan with no input from municipal hospitals and healthcare providers. Second, the monitoring of quality metrics negatively impacts community healthcare centers, creates administrative burden with center managers under continuous pressure to meet the metrics and secure the primary physician annual salaries. In their reports, stakeholders stated that several measures were not directly applicable to their center, but still had to meet the targets to maintain center ranking and continue to provide care. Three alternatives and opportunities are discussed to address the gaps and advance diabetes quality measurement:

- Revise and update the current quality measures – An alternative to the top-down process of measure development and implementation would be to make available opportunities for public comments. Such opportunities are key for reviewing and updating the measures. Without well-designed and applicable measures, the quality of care cannot be properly evaluated. The National Quality Forum^5^ provides a set of five questions to guide public comments: (*a*) Does the measure contribute to the overall goals and objectives of the program? (*b*) Does the measure result in better patient outcomes? (*c*) Does the measure reflect the current evidence? (*d*) Is there a high level of reporting burden for reporting entities? (*e*) Does the measure have negative unintended consequences, including impacts to the rural population or contribution to health disparities? In this particular research, there is a need to offer community health centers, patients, doctors and administrators the opportunity of comments on measures under development, and to provide feedback on measures currently implemented. Quality measurement is a continuous process, and the engagement of stakeholders is vital to its implementation and improvement as well as sustainability. It is possible that the engagement of the community can help increase the patient volume in the primary care system, alleviating the demand on secondary and tertiary centers.
- The need for precise documentation and collaboration – Given the complexity of quality measurement, healthcare organizations, providers, policy-makers need to work closely to develop, implement, and refine quality measures. There is also a need to ensure that the collection of data is accurate and comprehensive. It is essential that modern electronic health records and data infrastructure are available to ensure precise data collection and reporting, and avoid possible gaps in measurement. The advantages and disadvantages of the pay-for-performance system are listed with important ethical and practical issues raised, particularly the falsification of data. Regardless of the objective of the quality measurement, it requires accurate, current and actionable data. If the wrong outcomes are being measured, we will not know if there are improved outcomes or improved processes of care.
- Proliferation of quality metrics – the number of diabetes quality measures increased sharply in the United States for the past 25 years, with limited impact in advancing the quality of care or patient health outcomes.^6^ As quality measurements receives increased attention in China and other parts of the world, there is a need to control and focus on standard, valid and meaningful sets of measures that can address guidelines by professional medical organizations and initiatives as the Healthy People 2030 in the United States^7^ and Healthy China 2030.^8^

## Next Steps

 In their research, the authors provide a qualitative overview evaluating the impact of primary care quality measurement in Shanghai, China, where healthcare system policies changed in the past decades including increased coverage and the impact of such changes in patient behaviors is still being evaluated. As quality evaluation expands in healthcare worldwide, there are lessons to be learned from the barriers and facilitators identified in this paper that could be applied or replicated in other cities and provinces in China and internationally.

 The biggest limitation of this research is that it was done in one metropolitan area, and as the authors pointed out, it is unclear to which extent the findings from this evaluation are generalizable to other large or mid-size cities, or rural areas, as they may experience other barriers and facilitators. Quality measures need to account for the diverse populations they evaluate. Diverse patient demographics, cultural aspects, and socioeconomic factors can influence appropriateness and effectiveness of care.

 It would have been informative, to have a discussion on the potential of participant and researcher bias inherent to qualitative evaluations. For example, the selection of policy-makers was based on a convenience sample, and could increase the likelihood of selection bias. The likelihood of friendliness and social desirability bias where respondents feel the need to provide a positive spin on answers that were more critical of a centralized system cannot be ruled out.

 (1) To understand patients perspectives on the primary healthcare in China, Wang et al^9^ conducted qualitative research with 142 patient interviews around the same time as the Rasooly et al research but in a different city in China. This article showed that the distrust in the primary healthcare in China is the main driver of patients avoiding primary care and suggested that additional funds coming from private investments could help alleviate the crisis. (2) The stress level of doctors, managers and policy-makers at CHCs is an important consideration. Although the good intentions of the primary care doctors and staff at CHCs are noticeable, it was not explored in this research if the pressure imposed by a centralized system in fact decreased performance, especially as related to patients with more advanced disease. In general, response analysis showed there was alignment among the stakeholders about the barriers, where improvement is needed and that better collaboration between CHCs and tertiary care can be beneficial for providers, patients and the healthcare system.

## Conclusions

 Quality and performance measurement in primary diabetes care are important aspects of healthcare delivery not only in China but also around the world. Diabetes is a global health concern as the world population increases, ages, and struggles to maintain an active and healthy lifestyle.

 This research shows the importance of continuously monitoring and updating quality measures. Allowing stakeholders to express their views is crucial, and the methodology employed in this study can be systematically replicated by other researchers to identify gaps in the quality measures and patient-centered care and ultimately to improve care for people with diabetes. As China is moving towards national quality measurement in primary care, there is a need to revamp the current quality evaluation structure of diabetes care to better align with clinical guidelines best practices to ensure adherence to evidence-based care and meaningfully leading to improved care for the people with diabetes.

## Acknowledgements

 I would link to thank Dr. Gary Frey, DDS Professor and Chairman of the Department of General Practice and Dental Public Health and Dr. Muhammad Walji, PhD Professor and Associate Dean for Department of Clinical and Health Informatics at McWilliams School of Biomedical Informatics.

## Ethical issues

 Not applicable.

## Competing interests

 Author declares that she has no competing interests.
